# New recommendations on cerebral venous and dural sinus thrombosis from the German consensus-based (S2k) guideline

**DOI:** 10.1186/s42466-024-00320-9

**Published:** 2024-04-19

**Authors:** C Weimar, J Beyer-Westendorf, FO Bohmann, G Hahn, S Halimeh, S Holzhauer, C Kalka, M Knoflach, H-C Koennecke, F Masuhr, M-L Mono, U Nowak-Göttl, E Scherret, M Schlamann, B Linnemann

**Affiliations:** 1 BDH Klinik Elzach und Institut für Medizinische Informatik, Biometrie und Epidemiologie, Universitätsklinikum Essen, Essen, Germany; 2 Department of Medicine I; Division “Thrombosis & Hemostasis “, Dresden University Hospital „Carl Gustav Caris; Technical University Dresden, Dresden, Germany; 3 Department of Neurology, University Hospital, Goethe University Frankfurt, Frankfurt, Germany; 4 Department of Pediatric Radiology, University Children`s Hospital Basel UKBB, Basel, Switzerland; 5 Universitätsklinikum Essen, gerinnungszentrum rhein-ruhr, Duisburg, Germany; 6 Klinik für Pädiatrie mit Schwerpunkt Onkologie und Hämatologie, Charité – Universitätsmedizin Berlin, Berlin, Germany; 7 Vascular Institute Central Switzerland, Aarau, Switzerland and University of Cologne, Cologne, Germany; 8 Department of Neurology, Medical University of Innsbruck, Innsbruck, Austria; 9 Klinik für Neurologie, Vivantes Klinikum im Friedrichshain, Berlin, Germany; 10 Abteilung für Neurologie, Bundeswehrkrankenhaus Berlin, Berlin, Germany; 11 Abteilung für Neurologie, Stadtspital Triemli, Zürich, Switzerland; 12 Gerinnungszentrum UKSH (Campus Kiel und Lübeck), Institut für Klinische Chemie, Kiel, Germany; 13 Klinik für Neurologie der Charité – , Universitätsmedizin Berlin, Berlin, Germany; 14 Sektion Neuroradiologie, Institut für Diagnostische und Interventionelle Radiologie, Klinikum der Universität zu Köln, Cologne, Germany; 15 Klinik für Kardiologie III – Angiologie, Universitätsmedizin Mainz, Mainz, Germany

**Keywords:** Cerebral venous thrombosis, Dural sinus, Cerebral vein, D dimers, Anticoagulation, Thrombectomy, Hemicraniotomy, Decompressive surgery, Contraception, Pregnancy

## Abstract

Over the last years, new evidence has accumulated on multiple aspects of diagnosis and management of cerebral venous and dural sinus thrombosis (CVT) including identification of new risk factors, studies on interventional treatment as well as treatment with direct oral anticoagulants. Based on the GRADE questions of the European Stroke Organization guideline on this topic, the new German guideline on CVT is a consensus between expert representatives of Austria, Germany and Switzerland. New recommendations include:

• CVT occurring in the first weeks after SARS-CoV-2 vaccination with vector vaccines may be associated with severe thrombocytopenia, indicating the presence of a prothrombotic immunogenic cause (Vaccine-induced immune thrombotic thrombocytopenia; VITT).

• D-dimer testing to rule out CVT cannot be recommended and should therefore not be routinely performed.

• Thrombophilia screening is not generally recommended in patients with CVT. It should be considered in young patients, in spontaneous CVT, in recurrent thrombosis and/or in case of a positive family history of venous thromboembolism, and if a change in therapy results from a positive finding.

• Patients with CVT should preferably be treated with low molecular weight heparine (LMWH) instead of unfractionated heparine in the acute phase.

• On an individual basis, endovascular recanalization in a neurointerventional center may be considered for patients who deteriorate under adequate anticoagulation.

• Despite the overall low level of evidence, surgical decompression should be performed in patients with CVT, parenchymal lesions (congestive edema and/or hemorrhage) and impending incarceration to prevent death.

• Following the acute phase, oral anticoagulation with direct oral anticoagulants instead of vitamin K antagonists should be given for 3 to 12 months to enhance recanalization and prevent recurrent CVT as well as extracerebral venous thrombosis.

• Women with previous CVT in connection with the use of combined hormonal contraceptives or pregnancy shall refrain from continuing or restarting contraception with oestrogen–progestagen combinations due to an increased risk of recurrence if anticoagulation is no longer used.

• Women with previous CVT and without contraindications should receive LMWH prophylaxis during pregnancy and for at least 6 weeks post partum.

Although the level of evidence supporting these recommendations is mostly low, evidence from deep venous thrombosis as well as current clinical experience can justify the new recommendations.

This article is an abridged translation of the German guideline, which is available online.

## Introduction

This article is an abridged translation of the German guideline, which is available online [1].

A complete version of this guideline (in German) can be found on the website of the Deutsche Gesellschaft für Neurologie (https://dgn.org/leitlinien) and the AWMF (Arbeitsgemeinschaft wissenschaftlicher Medizinischer Gesellschaften, https://register.awmf.org/de/leitlinien).

For the current German guideline, key questions were based on the Grading of Recommendations, Assessment, Development, and Evaluation (GRADE) questions published by the European Stroke Organization (ESO) guideline in 2017 [2]. Therefore, only new or updated recommendations are presented in this overview.

## Definition

CVT is defined as a rare form of stroke where thrombosis occurs in the venous side of the brain circulation leading to obstruction of one or more cerebral veins and / or dural venous sinus.

## Methods of guideline development

This guideline is registered with www.awmf.org under 030/098.

Level of evidence: S2k.

Date of last update: 24.10.2023.

Valid until: 23.10.2023.

Edited by: Deutsche Gesellschaft für Neurologie e. V. (DGN).

Joint recommendations / Societies participating in guideline development:

- Deutsche Schlaganfall-Gesellschaft e.V. (DSG)
- Deutsche Gesellschaft für Neuroradiologie e.V. (DGNR)
- Deutsche Gesellschaft für Kinder- und Jugendmedizin e.V. (DGKJ)
- Deutsche Gesellschaft für Angiologie-Gesellschaft für Gefäßmedizin e.V. (DGA)
- Gesellschaft für Thrombose und Hämostaseforschung e. V. (GTH)
- Gesellschaft für Pädiatrische Radiologie e. V. (GPR)
- Österreichische Gesellschaft für Neurologie (ÖGN)
- Österreichische Schlaganfall-Gesellschaft (ÖGSF)
- Schweizerische Neurologische Gesellschaft (SNG)

We aimed to update the ESO guideline published in 2017 using its existing GRADE questions. To this end, a selective literature review was perfomed to cover the time since last systematic review performed by the ESO guideline panel. All topics were worked on by author-teams and then coordinated in a first Delphi round by the guideline group.

A strong recommendation corresponds in the formulation to a “shall”, a moderate recommendation to a “should” and an open recommendation to a “can”. In a second Delphi round, all recommendations were finally agreed upon by the guidelines group.

Based on this expert consensus, the formulation of the core statements was evaluated as strong agreement in the case of > 95% of all experts, as moderate agreement in the case of  > 75–95%, as majority agreement in the case of > 50–75%, and as no agreement in the case of ≤ 50%. In this abbreviated guideline we only refer to agreements of 90–100%.

The guideline was reviewed by the Guideline Committee of the German Neurological Society (DGN) and approved by the DGN. Interdisciplinarity was established. Patient organizations were not involved.

All participants in the guideline have submitted their declarations of interest (AWMF form for the declaration of interests in the context of guideline projects) to the coordinator and the Editorial Office for Guidelines of the DGN in time and completely filled out. The evaluation of the declarations of interest with regard to thematic relevance to the guideline was carried out by CW and the Editorial Office for Guidelines of the DGN. An external evaluation of the declarations of interest of CW was carried out by an independent reviewer of the DGN. Less than 50% of authors reported potential conflicts of interest for direct oral anticoagulants and abstined from the vote on the respective recommendation. For reasons of transparency, the interests of the participants and the consequences drawn therefrom are listed on the respective AWMF guideline website.

This guideline has been produced without any influence or support from industry and is provided by the authors free of charge.

## Preliminary note

In patients with septic CVT, the initial focus is on eliminating the focus of infection and on antibiotic treatment, which is not part of this guideline. Otherwise, treatment does not differ from aseptic CVT, and therefore septic CVT is not discussed separately.

## Diagnosis

If CVT is clinically suspected, cerebral imaging is the most important diagnostic test. Digital subtraction angiography is only rarely indicated if the other imaging procedures do not provide congruent findings. Otherwise, it plays practically no role in the diagnosis of CVT today.

*Key question: In patients suspected of CVT, should computed tomography with venous angiography versus magnetic resonance imaging with venous angiography be used to diagnose CVT?
*

**Table:** 

Strong Recommendation	modified 2023
Computed tomography (CT) and magnetic resonance imaging (MRI), each with venous angiography, are considered equivalent in the diagnosis of sinus thrombosis. MRI is superior to CT for cortical venous thrombosis. Due to the lack of radiation exposure, MRI shall be preferred in younger patients and during pregnancy	
Moderate Consensus 93.3%	

*Key question: In patients suspected of acute CVT should D-dimer be measured before neuroimaging to diagnose CVT?
*

**Table:** 

Moderate Recommendation	new 2023
D-dimer testing to rule out CVT cannot be recommended and should therefore not be routinely performed. Only in selected cases (low clinical probability with only isolated headache, lack of neurological symptoms and symptom duration < 30 days), negative D-dimers have a sufficiently reliable negative predictive value to justify the omission of neuroimaging	
Strong Consensus 100%	

*Key question: In patients with CVT, does a policy of screening for thrombophilia prevent recurrent venous thrombosis, reduce death and improve functional outcome?
*

**Table:** 

Moderate Recommendation	new 2023
General thrombophilia screening with the aim of reducing mortality or improving functional outcome cannot be recommended. In analogy to the procedure for lower-extremity deep vein thrombosis and pulmonary embolism, knowledge of thrombophilia can influence the decision on the type and duration of anticoagulation in individual cases and should therefore be considered preferably in young patients, in spontaneous CVT, in recurrent thrombosis and/or in patients with a positive family history of venous thromboembolism, and if therapeutic consequences can be derived from the result	
Moderate Consensus 93.3%	

Even if there is a lack of study data proving a benefit of etiologic workup, it is still useful to identify the relevant risk factors suspected to have contributed to the thrombosis in every patient with CVT, as this can have an influence on treatment and secondary prophylaxis. The most common risk factors or causes of CVT include [3]:

- Female gender (75%)
- Combined hormonal contraceptives (40–50% of all women with CVT)
- Thrombophilia (34–40%)
- Pregnancy (especially in the 3rd trimester) and postpartum phase (10–20%)
- Local infections, e.g. otitis, mastoiditis, sinusitis, stomatitis, dental abscesses, meningitis, brain abscess (≈10%)
- JAK2V617F mutation (6–7%)
- Myeloproliferative disease (4%)
- Hormone replacement therapy (3–4%)
- Cerebral neoplasia (≈2%)
- Idiopathic (10–20%)

Other rare risk factors or comorbidities:

- Disseminated intravascular coagulation
- Paroxysmal nocturnal hemoglobinuria (PNH)
- Heparin-induced thrombocytopenia
- Vaccine-induced prothrombotic immunogenic thrombocytopenia (VITT)
- Severe hyperhomocysteinemia
- Dysfibrinogenemia
- Malignancies: carcinomas, lymphomas, carcinoid, leukemias
- Sickle cell anemia, hypochromic or hemolytic anemia
- Collagenoses: lupus erythematosus, Sjögren's syndrome
- Vasculitides: Behçet's disease, granulomatosis with polyangiitis (GPA; formerly: Wegener's granulomatosis)
- Sarcoidosis

Very rare causes are

- Intracranial hypotension (cerebrospinal fluid hypotension syndrome)
- Lumbar cerebrospinal fluid puncture: CVT can occur with a time delay after a cerebrospinal fluid puncture. In these cases, in contrast to CSF hypotension syndrome, the headache usually increases when lying down.
- Local: craniocerebral trauma, neurosurgical operations, mechanical outflow obstruction due to tumors
- Disorders with venous stasis: central venous catheters, strangulation, dural arteriovenous malformation
- Drug-related toxic causes: Androgens, chemotherapeutic agents, corticosteroids, erythropoietin, vitamin A overdose, E. coli-derived asparaginase in combination with prednisone, illegal drugs
- Metabolic diseases: Diabetes mellitus, thyrotoxicosis, uremia, nephrotic syndrome
- Gastrointestinal tract diseases: liver cirrhosis, Crohn's disease, ulcerative colitis
- Cardiac diseases: heart failure, cardiomyopathy

Generalized infectious diseases:

- Bacterial: septicemia, endocarditis, typhoid fever, tuberculosis
- Viral: measles, hepatitis, encephalitis (HSV, HIV), cytomegalovirus
- Parasitic: malaria, trichinosis
- Fungal infections: Aspergillosis

For children with CVT, the causes listed in the literature are [3]:

- Thrombophilia
- Dehydration
- Perinatal complications
- Infections
- Solid tumors
- Hemato-oncological diseases

In children and adolescents with venous thrombosis, including CVT, the correlation between the initial manifestation of thrombosis or thromboembolic recurrences and congenital thrombophilic risk factors is more pronounced than in adults [4, 5].

## Therapy

**Fig. 1:**
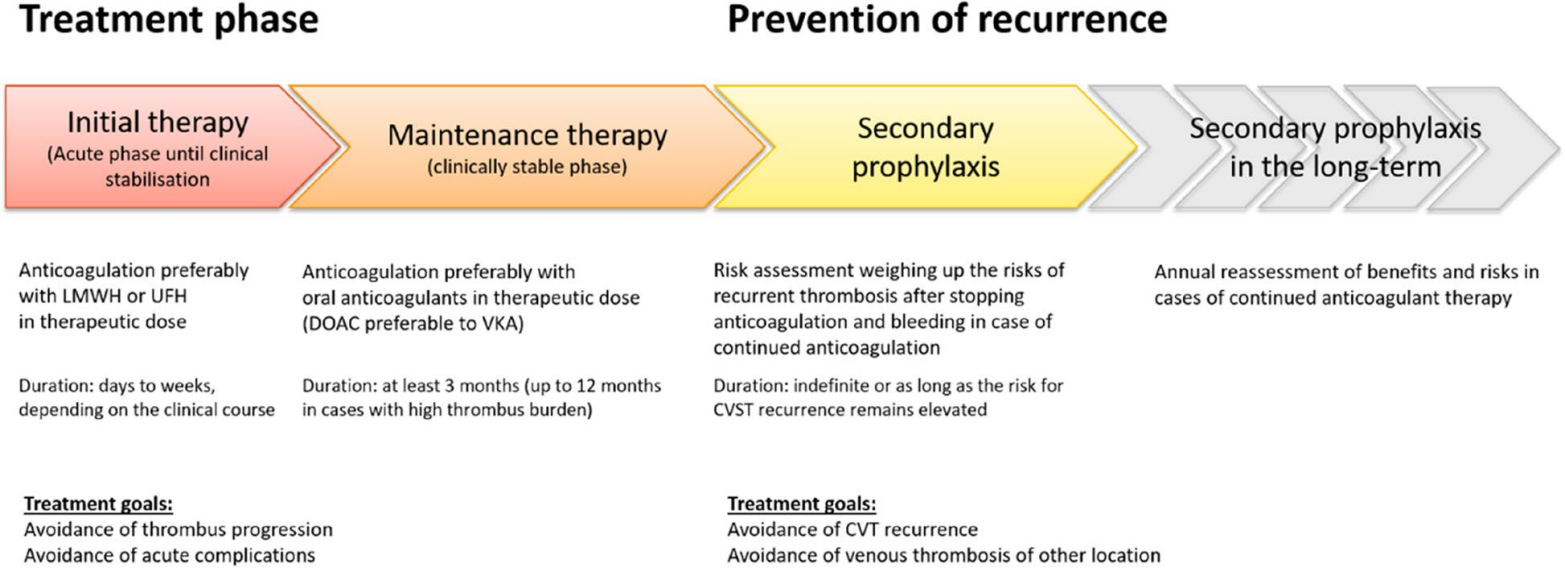
Phases in the treatment of CVT

Anticoagulation in the acute phase of CVT is essential to prevent propagation of the thrombus or renewed thrombotic occlusion of vessel sections that have already been reopened by the body's own fibrinolysis. Anticoagulation is also indicated in case of intracranial bleeding due to venous congestion from CVT. There are 3 treatment phases in anticoagulant therapy like for venous thrombosis (Fig. 1) [6]. Treatment is usually initiated with parenteral anticoagulation (initial phase) before switching to a maintenance regimen, usually with an oral anticoagulant. At the end of maintenance phase, anticoagulation is either discontinued or, only in case of an increased risk of recurrence, continued as secondary prophylaxis.

### Anticoagulation in the acute phase

During the acute phase, treatment should take place under monitoring conditions in a stroke unit (or intensive care unit if necessary) in order to recognize and treat any clinical deterioration or complications at an early stage. A transfer to a center with interventional neuroradiology and neurosurgery is recommended at the latest when signs of intracranial pressure appear. Due to individual courses with more or less pronounced neurological symptoms and varying risks of bleeding and recurrence, the acute phase is not defined in terms of time. Therefore, stable or oligosymptomatic (e.g. isolated headache) patients without evidence of bleeding can be treated like in maintenance therapy, on a normal ward or primarily on an outpatient basis.

*Key question: In patients with acute CVT, does anticoagulation improve clinical outcome compared to no anticoagulation?
*

**Table:** 

Strong Recommendation	Modified 2023
Patients with CVT shall be treated with therapeutic doses of heparin in the acute phase, regardless of whether intracranial hemorrhage is present	
Strong Consensus 100%	

*Key question: In patients with acute CVT does low molecular weight heparine (LMWH) improve clinical outcome compared to unfractionated heparine (UFH)?
*

**Table:** 

Moderate Recommendation	Modified 2023
Patients with CVT in the acute phase should preferably be treated with LMWH over UFH UFH should be given preference before an operative or endovascular intervention or in case of contraindications to LMWH	
Strong Consensus 100%	

As treatment with LMWH does not require intravenous access or regular laboratory checks, it is also preferable in practical use, as it has been shown to be more effective and safer than UFH in individual studies and meta-analyses [7, 8]. Advantages are the easier application through once or twice daily subcutaneous injections [9], the lack of need for laboratory monitoring and frequent dose adjustments as well as a lower risk of developing heparin-induced thrombocytopenia (HIT) type II. The European guideline on the treatment of CVT therefore recommends treatment with weight-adapted LMWH, as does the AWMF S2k guideline on the diagnosis and treatment of venous thrombosis and pulmonary embolism [2, 6]. There is a significant restriction on the use of LMWH in patients with advanced renal insufficiency.

However, the overall evidence regarding the comparison of LMWH and UFH in patients with CVT is low. The randomized trial and prospective observational study presented were associated with a high risk of bias and a systematic literature review with meta-analysis of three studies (179 patients under LMWH, 352 patients under UFH) failed to demonstrate a significant difference in mortality or functional outcome [10]. UFH has a possible advantage in patients requiring intensive care who may require short-term surgical intervention, as coagulation normalizes within one to two hours after the end of intravenous heparin therapy. Patients who have a contraindication to the use of LMWH (severe renal insufficiency) should also be treated with UFH.

There are currently no randomized studies on the use of direct oral anticoagulants (DOAC) in the acute phase of CVT. In contrast to the treatment of VTE, no recommendation can therefore currently be made on their use in the acute phase of CVT. Instead, clinical stabilization under heparin treatment should be awaited first.

### Thrombectomy und Thrombolysis

Neuroradiological interventional therapy is based on transvenous mechanical thrombectomy using stent retrievers and/or aspiration catheters. Transarterial lysis therapy is no longer performed. At the discretion of the neurointerventionalist, local (catheter-directed) thrombolysis may be used as an adjunct to mechanical thrombectomy in individual cases.

*Key question: Does endovascular recanalization improve clinical outcome compared to anticoagulation in patients with acute cerebral venous thrombosis?
*

**Table:** 

Weak Recommendation	New 2023
In individual cases, endovascular recanalization in a neurointerventional center can be considered for patients who deteriorate under adequate anticoagulation	
Strong Consensus 100%	

A randomized trial (TO-ACT) in patients at high risk of poor clinical outcome (defined as at least one of the following risk factors: qualitative or quantitative impairment of consciousness, parenchymal hemorrhage or deep cerebral vein thrombosis) was terminated prematurely and showed no benefit of endovascular thrombolysis with or without thrombectomy versus therapeutic anticoagulation [11]. The other data published to date are based on retrospective and uncontrolled case series and mostly examined local thrombolysis with alteplase or urokinase, in some cases with thrombectomy by aspiration, stent retriever thrombectomy, or balloon-guided thrombectomy/angioplasty with stent placement [12]. A meta-analysis was unable to demonstrate any difference between the procedures used [13].

Thus, although local thrombolysis alone or in combination with thrombectomy has high recanalization rates, it is associated with higher overall bleeding complications, with no evidence of improved clinical outcome to date. Patients with CVT who have a low risk of poor clinical outcome are therefore unlikely to be suitable candidates for local thrombolysis. In patients with large space-occupying hemorrhagic infarcts, thrombolytic therapy can lead to an increase in the size of the hemorrhage and thus accelerate the process of impending entrapment.

In individual cases, endovascular recanalization, either as venous thrombectomy alone or in combination with thrombolysis, can be considered as an individual treatment attempt in a specialized (neurointerventional) center for other patients who deteriorate under adequate anticoagulation.

### Intracranial pressure therapy

Although cerebral edema can be detected on imaging in up to 50% of all patients with CVT, specific measures to reduce intracranial pressure are only necessary in a small number of cases.

*Key question: For patients with acute CVT and parenchymal lesion(s) with impending herniation, does decompressive surgery (hemicraniectomy or haematoma evacuation) improve outcome, compared with conservative treatment?
*

**Table:** 

Moderate Recommendation	New 2023
Despite the overall low level of evidence, surgical decompression should be performed in patients with acute CVT, parenchymal lesions (congestive edema and/or hemorrhage) and impending incarceration to prevent death	
Strong Consensus 100%	

There are no current or anticipated randomized studies on this question, but several partly controlled case series and current reviews [14, 15]. Even if the overall number of cases is small, the results consistently indicate that deaths can be prevented by means of surgical decompression in the event of imminent incarceration without simultaneously leading to an increase in the proportion of severely disabled patients. In addition, some cases are reported in which a good functional result was achieved despite advanced clinical signs of herniation.

### Anticoagulation in the maintenance phase

The duration of the acute phase of CVT varies, and the associated decision for anticoagulation must be made on an individual basis and depending on the patient's symptoms and complications. Maintenance therapy therefore begins after clinical stabilization and usually after monitoring phase. At this point, the aim in everyday clinical practice is to switch from parenteral to oral anticoagulation. DOAC and vitamin K antagonists (VKA) are available for this purpose.

*Key question: In patients with cerebral venous thrombosis, does treatment with DOAC improve clinical outcome, reduce major haemorrhagic complications and reduce thrombotic recurrences, compared to conventional anticoagulation (heparin and VKA)?
*

**Table:** 

Moderate Recommendation	New 2023
DOAC should be preferred over VKA for oral anticoagulation of CVT patients. Exceptions are patients with triple-positive antiphospholipid syndrome, for whom VKA therapy should be preferred	
Strong Consensus 100%	

In large studies in patients with atrial fibrillation or lower-extremity deep vein thrombosis and pulmonary embolism, DOAC have shown a significantly better safety profile compared to VKA with comparable efficacy.

In addition to numerous prospective and retrospective observational studies, the results of two randomized studies are now available comparing DOAC with VKA in CVT [16, 17]. In a meta-analysis of these two studies, the risk ratio (RR) for any VTE complication was 0.17 (95% CI 0.02–1.71) and for CVT recurrence 0.37 (95% CI 0.03–4.3) in favor of DOAC therapy [18]. At the same time, the meta-analysis showed a trend towards a reduction in major bleeding (RR 0.35; 95% CI 0.05–2.36). The rate of complete CVT recanalization was comparable in both arms (RR 1.08; 95% CI 0.98–1.82).

In the same meta-analysis, 15 non-randomized retrospective and prospective observational studies of varying cohort size and study quality were also evaluated. There was a tendency towards comparable observations to the RCTs with regard to the rate of thromboembolic complications (all VTE: RR 1.2; 95% CI 0.48–2.98; CVT recurrences: RR 1.57; 95% CI 0.5–4.92), with regard to complete recanalization (RR 1.45; 95% CI 0.97–2.17) and the risk of major bleeding (RR 0.6; 95% CI 0.2–1.79).

Neither the two randomized trials nor the pooled observational studies showed a disadvantage for DOAC therapy with regard to the modified Rankin Scale or mortality. Overall, the available data on DOAC therapy in CVT confirm its efficacy and safety. Based on the data, but also due to the higher patient acceptance, DOAC should therefore be preferred in maintenance therapy, with the evidence for dabigatran being the best in adults with CVT.

Anticoagulant therapy with LMWH in children with CVT beyond the acute phase has been investigated in numerous observational studies and a subgroup of a randomized study over a period of between 3 and 6 months without significantly associated bleeding and/or rethrombotic events [4, 19–23]. This results in the recommendation that LMWH can be used for short-term anticoagulant therapy for a median of 3 months in children with CVT. Results from the randomized EINSTEIN-Junior study suggest that rivaroxaban is not inferior to this standard therapy. Other DOAC (apixaban, dabigatran, edoxaban) have not yet been tested in pediatric CVT trials.

When selecting a suitable oral anticoagulation for CVT patients, it should be noted that data in patients with adequately confirmed triple-positive antiphospholipid syndrome (APS; positive for lupus anticoagulant, cardiolipin IgG and ß2-glycoprotein IgG) speak against the use of DOAC in VTE patients [24, 25]. Even if there are no studies on patients with CVT, the transfer of these study results should also lead to a preference for VKA in CVT patients with triple-positive APS.

*Key question: For patients with CVT, does treatment with long-term anticoagulation (> 6 months) improve outcome, compared with treatment with short-term anticoagulation (< 6 months)?
*

**Table:** 

Strong / Moderate Recommendation	New 2023
In CVT patients, the duration of anticoagulation shall not be less than 3 months. The duration of anticoagulation should not be extended beyond the maintenance therapy phase (3 to 12 months) with the sole aim of achieving further recanalization or an improvement in the clinical outcome of the index event	
Strong Consensus 100%	

Recanalization rates after CVT were examined in a systematic review [26] which showed a recanalization rate of 85% (95% CI 80–89%) in 818 CVT patients from 19 studies and a recanalization rate of 77% (95% CI 70–825) in the studies with high methodological quality.

Available studies also show that the majority of recanalization takes place in the first 3–6 months [27] and that hardly any further recanalization occurs after > 12 months [28–30].

*Key question: For patients with previous CVT, does treatment with long-term anticoagulation reduce recurrence of venous thrombotic events, compared with treatment with short-term anticoagulation?
*

**Table:** 

Strong / moderate Recommendation	New 2023
In CVT patients with a low risk of recurrent CVT or extracerebral venous thrombosis, anticoagulation shall be discontinued after 3 (to 12) months. In CVT patients with an increased risk of recurrent CVT or extracerebral venous thrombosis, anticoagulation should be continued in the long term as secondary prophylaxis to prevent VTE recurrences	
Consensus 100%	

There are no evidence-based CVT-specific criteria for assessing the individual VTE recurrence risk. It is therefore recommended to proceed analogously to the considerations for extracranial VTE manifestations [6]. The first step should be to identify and assess the possible trigger of the index CVT. If there was a clear trigger (infection, hormone therapy, etc.) and this has since been eliminated, the risk of VTE recurrence is considered to be acceptably low to justify discontinuation of anticoagulation. However, if the trigger persists (e.g. malignancy, severe thrombophilia, active autoimmune diseases, etc.), an increased risk of recurrence should be assumed and prolonged secondary prophylaxis should be considered in individual cases for longer than 12 months, especially if anticoagulation is well tolerated, the risk of bleeding is low and the patient is more likely to continue anticoagulation.

It is advisable to repeat this risk–benefit assessment regularly if secondary prophylaxis is continued in order to recognize a change in the risk profile and to adjust the anticoagulation accordingly. This also includes the anticoagulation intensity for secondary prophylaxis. As there is no randomized study on this question in CVT patients, the dosage of continued secondary prophylaxis can be selected in analogy to the considerations for extracranial VTE manifestations [6].

### Contraception and Pregnancy issues in CVT patients

*Key question: In pregnant and puerperal women with CVT, does the use of anticoagulant therapy improve the outcome without causing major risks to mother and foetus?
*

**Table:** 

Moderate Recommendation	New 2023
CVT occurring during pregnancy or the postpartum period should be treated with subcutaneously administered LMWH in therapeutic doses	
Strong Consensus 100%	

To date, no controlled randomized therapy studies on CVT during pregnancy and the postpartum period have been published. Nevertheless, it can be assumed that anticoagulation improves the prognosis for pregnant women like for non-pregnant women in the acute phase, especially as physiological pregnancy-related hypercoagulability contributes to the occurrence of thrombosis. The evidence from observational studies and small case series of pregnant women was compiled in the ESO guideline [2]. There was evidence of a better clinical prognosis under anticoagulation with LMWH and the risk of bleeding complications with LMWH therapy was very low [31–34]. As heparins do not cross the placenta barrier, they can be used without risk to the fetus. Heparins are therefore the drugs of choice if anticoagulation is required during pregnancy.

DOAC and VKA, on the other hand, can pass the placenta to the foetus, which is why they should not be used to treat CVT during pregnancy.

Studies on the optimal duration of therapy are not available. Due to the increasing risk of VTE during the course of pregnancy and a maximum in the early postpartum phase, anticoagulation is recommended until at least 6 weeks post partum in analogy to lower-extremity deep vein thrombosis and pulmonary embolism [35] whereby the total duration of treatment should not be less than 3 months. The peripartum management of women with CVT should ideally be carried out by a multidisciplinary team with hemostasis expertise.

In addition to LMWH, fondaparinux and VKA are also considered safe during breastfeeding. During VKA therapy, care should be taken to ensure that newborns have an adequate vitamin K intake in the first weeks of life (www.embryotox.de). The use of DOACs during breastfeeding is not recommended, as the extent to which these substances or their metabolites pass into breast milk has not been sufficiently investigated [36].

*Key question: In women with prior CVT does use of combined oral hormonal contraception increase the risk of recurrent CVT or other VTE?
*

**Table:** 

Strong Recommendation	New 2023
Women with previous CVT in connection with the use of combined hormonal contraceptives or pregnancy shall refrain from continuing or restarting contraception with oestrogen–progestagen combinations due to an increased risk of recurrence if anticoagulation is no longer used	
Strong Consensus 100%	

In unselected collectives, the recurrence rate for CVT in the first year after stopping anticoagulation is 2–5% [37–39]. According to a systematic review, the use of combined hormonal contraceptives is associated with a 7.6-fold increased risk (95% CI 3.82–15.09) of suffering a CVT [40]. The increased risk of venous thromboembolism depends on the oestrogen content and the progestogen component in combination products. Oral progestagen monopreparations or intrauterine devices containing levonorgestrel, on the other hand, do not increase the risk of thrombosis and can therefore be used safely even after previous VTE or CVT. There is insufficient experience with depot preparations in VTE patients—they should therefore be avoided.

The current AWMF S3 guideline on hormonal contraception calls for effective contraception for women of childbearing age undergoing treatment with an oral anticoagulant to avoid unplanned pregnancies and the associated risks (e.g. thromboembolism, embryonic malformations) [41–43]. In women with proximal deep venous thrombosis and/or pulmonary embolism, combined hormonal contraception can initially be continued under therapeutic dosing. A subgroup analysis of the EINSTEIN-DVT/-PE study with women before the age of 60 has shown that the continuation of hormone therapy with combined oestrogen–progestagen preparations under the protection of therapeutically dosed anticoagulation is not associated with an increased risk of VTE recurrence compared to women who do not take hormones [44].

However, continued combined hormonal contraception should be discontinued at least 6 weeks before the planned discontinuation of anticoagulation. Even if no corresponding data are available for women with CVT, it seems justified to transfer this established procedure for classic VTE to CVT. In women with a history of CVT who have stopped anticoagulation, an estrogen-free contraceptive method should be chosen to minimize the risk of recurrent CVT.

Women with CVT and ongoing anticoagulant therapy should be informed about the risks of unintended pregnancy and the need for effective contraception under oral anticoagulation. Women with a history of CVT and after discontinuation of anticoagulation should be informed about the increased risk of recurrence when taking an oestrogen-progestogen combination for hormonal contraception again or during perimenopausal hormone replacement therapy.

*Key question: In females with previous history of CVT is the risk of pregnancy-related CVT recurrence or other VTEs (lower or upper extremity deep vein thrombosis, pulmonary embolism, abdominal or pelvic venous thrombosis) increased?
*

**Table:** 

Moderate Recommendation	New 2023
The extent to which pregnancy increases the risk of recurrent CVT has not been adequately investigated. Women should be advised that the risk of recurrent CVT or other VTE is low if no additional VTE risk factors are present	
Moderate Consensus 93.3%	

There are several cohort studies with a low number of cases that analyzed the prognosis of pregnant women with previous CVT [45–49]. In these studies, the risk of recurrence was low, at 0–1.2%, although it should be noted that the majority of women with a history of CVT received secondary prophylaxis with LMWH (83–100%). The risk of other VTE occurring during pregnancy was 0–3.2%. Serious bleeding complications were not reported. However, the individual studies lacked a control group; therefore, the risk of recurrence in pregnant vs. non-pregnant women and in women with vs. without LMWH prophylaxis cannot be assessed.

*Key question: For pregnant women with previous history of CVT, does prophylaxis with antithrombotic drugs during pregnancy reduce the risk of thromboembolic events or affect pregnancy outcome?
*

**Table:** 

Moderate Recommendation	New 2023
In view of the low rates of recurrent CVT or other VTE during LMWH prophylaxis, this should be considered during pregnancy and for at least 6 weeks post partum, in analogy to other VTE, provided there are no contraindications. An indication for LMWH prophylaxis during pregnancy and the postpartum period exists in particular if CVT occurred spontaneously or in connection with a previous pregnancy or under combined hormonal contraception or if additional VTE risk factors are present. This should be taken into account when giving advice in the context of a planned pregnancy	
Strong Consensus 100%	

Due to a lack of study data, this question cannot be answered with certainty. The recommendations made here are therefore based on those for VTE prophylaxis in pregnant women after previous deep vein thrombosis and/or pulmonary embolism [50]. As pregnancy is physiologically a state of hypercoagulability, it can be assumed that the risk of CVT recurrence increases during the course of pregnancy in a similar way to the risk of VTE and is also increased in the first weeks post partum. A Cochrane review from 2021, which analyzed the risk and benefit of LMWH prophylaxis in pregnant women with an increased risk of VTE, concludes that there is insufficient data to prove a general benefit of LMWH prophylaxis [51]. Based on weak evidence, secondary prophylaxis with an LMWH at a dose approved for high-risk prophylaxis should be considered for women with previous hormone- or pregnancy-associated CVT for the duration of pregnancy and up to 6 weeks post partum, provided there are no contraindications.

### Prophylaxis in other risk situations

For adults with previous CVT, the corresponding recommendation of the AWMF S3 guideline on the prophylaxis of venous thromboembolism applies to the group with a high risk of venous thromboembolism, i.e. in risk situations, They should receive drug prophylaxis with an anticoagulant [52].

Children and adolescents who have already suffered a CVT should receive thromboembolism prophylaxis with weight-adapted, low-molecular-weight heparin in risk situations, such as immobilization > 4 days, rheumatic and oncological diseases, repeated exposure to E. coli, asparaginase and steroids, central venous catheter placement, air travel > 4 h [53].

## Data Availability

Not applicable.

## Abbreviations

CI: Confidence intervall
CT: Computed tomography
CVT: Cerebral venous and dural sinus thrombosis
DOAK: Direct oral anticoagulants
GRADE: Grading of Recommendations, Assessment, Development, and Evaluation
LMWH: Low molecular weight heparine
MRI: Magnetic resonance imaging
UFH: Unfractionated heparine
RR: Risk ratio
VKA: Vitamin-K-antagonists
VTE: Venous thromboembolic events
